# Guarding the Genome: CENP-A-Chromatin in Health and Cancer

**DOI:** 10.3390/genes11070810

**Published:** 2020-07-16

**Authors:** Megan A. Mahlke, Yael Nechemia-Arbely

**Affiliations:** 1 UPMC Hillman Cancer Center, Pittsburgh, PA 15213, USA; mahlkem@upmc.edu; 2 Department of Pharmacology and Chemical Biology, University of Pittsburgh, Pittsburgh, PA 15261, USA

**Keywords:** CENP-A, centromere, chromosome segregation, mitosis, epigenetic, kinetochore, CIN, cancer

## Abstract

Faithful chromosome segregation is essential for the maintenance of genomic integrity and requires functional centromeres. Centromeres are epigenetically defined by the histone H3 variant, centromere protein A (CENP-A). Here we highlight current knowledge regarding CENP-A-containing chromatin structure, specification of centromere identity, regulation of CENP-A deposition and possible contribution to cancer formation and/or progression. CENP-A overexpression is common among many cancers and predicts poor prognosis. Overexpression of CENP-A increases rates of CENP-A deposition ectopically at sites of high histone turnover, occluding CCCTC-binding factor (CTCF) binding. Ectopic CENP-A deposition leads to mitotic defects, centromere dysfunction and chromosomal instability (CIN), a hallmark of cancer. CENP-A overexpression is often accompanied by overexpression of its chaperone Holliday Junction Recognition Protein (HJURP), leading to epigenetic addiction in which increased levels of HJURP and CENP-A become necessary to support rapidly dividing p53 deficient cancer cells. Alterations in CENP-A posttranslational modifications are also linked to chromosome segregation errors and CIN. Collectively, CENP-A is pivotal to genomic stability through centromere maintenance, perturbation of which can lead to tumorigenesis.

## 1. Introduction

Equal chromosome segregation during mitosis is critical for ensuring genome stability and for successful transmission of the genetic material to the daughter cells. The centromere is the central genetic element responsible for faithful chromosome inheritance during mitosis. Loss of centromere identity and/or function can have detrimental consequences, including errors in chromosome segregation, in the form of misaligned and lagging chromosomes. Such chromosome segregation errors often lead to micronuclei formation and may result in aneuploidy, the presence of additional or fewer chromosomes. Sustained high levels of mitotic defects may ultimately lead to chromosomal instability (CIN), a condition prevalent among many cancers. Cancers are frequently aneuploid [1,2,3,4], and the specific loss or gain of tumor suppressors and oncogenes associated with changes in chromosome number may contribute to tumorigenesis, malignancy, frequency of metastasis, and overall patient prognosis [3,5,6]. In this review we will highlight recent advances in understanding centromere maintenance and propagation in health and tumorigenesis.

Known as the centromere paradox, centromeric DNA sequence is not conserved between species despite functional conservation [7]. In humans, the centromere is composed of extensive tandemly repeated arrays of a 171-bp DNA sequence element called α-satellite [8,9,10], organized into high-order repeat arrays (HOR), most of which can be uniquely assigned to specific chromosomes [10,11,12,13]. Centromeric α-satellite DNA sequences represent ~2–3% of the human genome [12,14,15], and can vary between individuals in a population through acquisition of rearrangements and/or repeat expansions [16]. Despite the fact that human centromeres are found in this unique and complex genomic location, α-satellite DNA sequences are neither sufficient nor essential for centromere identity [8,17], as evidenced by the growing numbers of identified neocentromeres. Neocentromeres are defined by the acquisition of a new centromere at a new location coupled with inactivation of the original centromere [18]. Neocentromeres lack α-satellite DNA but are epigenetically stable and can persist for several generations [18,19]. Centromere identity relies instead on epigenetic markers, with centromere protein A (CENP-A)-containing chromatin considered as the prime candidate, from yeast to human [20]. CENP-A is a centromere specific variant of the canonical histone H3, initially identified in humans [21,22], that marks, maintains and propagates centromere function indefinitely in human cells and fission yeast [23]. Knockout of CENP-A is embryonic lethal [24] and depletion or conditional knockout of CENP-A negatively impacts mitotic spindle pole integrity [25], resulting in increased rates of chromosome segregation errors [23,26].

As an epigenetic mark that identifies centromeres, tethering of CENP-A, or its depositing chaperone HJURP (Holliday Junction Recognition Protein), to a new genomic location can trigger local formation of a functional kinetochore in yeast [27], fly [28,29,30] and human [31,32], measured by recruitment of centromere and kinetochore proteins and by the ability to bind microtubules [28,31]. Studies in Human artificial chromosomes (HACs) have shown that HAC formation requires human α-satellite centromeric repeats, high density of CENP-B boxes [33,34] and CENP-B expression [35]. Logsdon and colleagues have recently improved HAC technology bypassing the need for α-satellite centromeric repeats and CENP-B boxes and instead using the epigenetic machinery to initiate centromere identity [32,36]. By tethering HJURP to a unique genomic location using the LacI—LacO system, they drove initial CENP-A nucleosome seeding, leading to stable centromere and HAC formation and resulting in a new generation of HACs built without α-satellite DNA [32].

## 2. The Structure and Composition of CENP-A-Containing Chromatin

CENP-A is a rare histone variant that represents only ~0.1% of the total histone H3 variants in the genome [37], while marking all active centromeres, including neocentromeres [17]. Centromeric chromatin is comprised of both CENP-A- and H3-containing nucleosomes [38,39]. While most (~97%) of the centromeric nucleosomes contain histone H3.1 [14], about 3–4% are assembled with CENP-A, representing ~200 CENP-A-containing nucleosomes per centromere on average [37].

The structure and composition of CENP-A-containing chromatin has been highly controversial (summarized in Black and Cleveland [40]) with prominent models including a conventional octameric nucleosome with 2 molecules each of CENP-A, H4, H2A and H2B [41,42] with a similar structure to histone H3-containing chromatin [43], a hemisome with only one molecule of each histone [44,45,46], a tetrasome lacking H2A and H2B [47], or a heterotypic nucleosome with one molecule of H3 and one molecule of CENP-A [48,49]. Another prominent model for CENP-A-containing chromatin suggested that CENP-A chromatin oscillates between two forms; a hemisome during G2, M and G1 transforming into an octameric nucleosome during S phase [50]. Advances made in recent years using a plethora of approaches have demonstrated that the overwhelming majority of CENP-A molecules assemble into homotypic, octameric nucleosomes, containing two molecules of CENP-A [51], H4, H2A and H2B [52] at all cell cycle points [14] with heterotypic CENP-A/histone H3-containing nucleosomes comprising at most 2% of CENP-A-containing chromatin [14]. The main feature that differentiates CENP-A- from H3-containing nucleosomes is the highly flexible DNA ends at the entry and exit sites of CENP-A-containing nucleosomes [43,53,54,55]. This is consistent with studies showing transient DNA unwrapping at these regions [48,56,57] at all cell cycle points [14], mediated by the CENP-A N-terminal tail and the CENP-A targeting domain (CATD) [14]. This change in chromatin structure may have a role in producing a more global condensed chromatin state [58] while loosening CENP-A nucleosome DNA superhelical termini and allowing CENP-A nucleosomes to be readily accessible for binding of centromeric proteins [43]. In accordance with this idea, several recent (cryogenic Electron Microscopy) cryo-EM studies of the CENP-A nucleosome demonstrated higher flexibility and open conformation of the DNA ends [55], leading to a less twisted conformation in tri-nucleosomes composed of a central CENP-A nucleosome linked to two H3 nucleosomes than in H3 tri-nucleosomes, and yielding a highly exposed CENP-A nucleosome, readily accessible for binding kinetochore components [59]. Moreover, linker histones were shown to bind weakly and occur rarely at CENP-A-containing chromatin [55,60], again increasing accessibility of CENP-A for binding of centromeric proteins. Interestingly, enhanced cryo-EM structural imaging of the CENP-A nucleosome core particle revealed a key difference between the two DNA termini: one end shows well defined density and associates closely with the histone octamer core, whereas the other appears flexibly disordered and partly unwrapped [54].

## 3. CENP-B Roles in Centromere Specification and Function

Some centromeric repetitive α-satellite DNA sequences contain a 17-bp motif [61] that serves as a binding site for CENP-B [21], the only sequence specific human centromere protein [61,62,63]. CENP-B plays a role in stabilizing centromere identity, through its direct interaction with both CENP-C and the N-terminal tail of CENP-A [23,64], as well as through its binding to the CENP-B box within α-satellite DNA [65]. These bindings sustain and stabilize centromere/kinetochore assembly during mitosis even when CENP-A is depleted [66]. Indeed, abundance of CENP-B at individual centromeres is associated with higher segregation fidelity in the absence of CENP-A [67]. Despite the contribution of CENP-B to the stability of endogenous centromeres [66,67], CENP-B is not essential for centromere function, as CENP-B boxes are absent from the human Y centromere, and neocentromeres lacking α-satellite DNA and CENP-B boxes are maintained through multiple cell divisions [68,69,70]. CENP-B is also absent from active centromeres at non-centromeric sites of dicentric chromosomes, and at centromeres of HACs lacking CENP-B boxes [17,19,32]. In addition, CENP-B knockout mice are viable [71,72,73] and CENP-B knockout cell lines can grow long-term [64], albeit with higher missegregation rates [64].

## 4. The Constitutive Centromere-Associated Network Role in Kinetochore Assembly

The centromeric chromatin provides a platform for binding of the constitutive centromere-associated network (CCAN) by adopting a higher order structure that partitions CENP-A nucleosomes towards the outer chromosome surface where they are maximally accessible for CCAN binding [18,59,74,75,76,77,78]. The CCAN is a large protein complex, consisting of 16 proteins, that links chromosomes and spindle microtubules by nucleating kinetochore assembly before mitotic entry. CCAN proteins are located in the inner kinetochore plate and distributed in several functional groups as follows: CENP-C, CENP-H/I/K, CENP-L/M/N, CENP-O/P/Q/R/U, CENP-T/W/S/X (for a detailed review see Hara and Fukagawa [79]).

Among the CCAN proteins, CENP-C is regarded as the blueprint for kinetochore assembly [80,81]. CENP-A uses its C-terminal tail [23] to bind CENP-C directly in two domains within CENP-C [82]. CENP-C binding to CENP-A stabilizes centromeric chromatin, reshapes the octameric histone core of CENP-A nucleosomes, rigidifies both surface and internal nucleosome structure, and unwraps the terminal DNA of CENP-A [83]. The unwrapping of the CENP-A nucleosome DNA ends facilitates further interaction with the CCAN [82]. Cyclin-dependent kinase (CDK)1-mediated phosphorylation of CENP-C facilitates its binding to CENP-A and CENP-A–CENP-C interaction is important for kinetochore localization of CENP-C during mitosis [84]. CENP-C serves as an anchor and a keystone protein that subsequently recruits other members of the CCAN, forming a stable platform for downstream kinetochore assembly [80,85,86,87,88,89]. Depletion of CENP-C results in loss of CENP-H/I/K/M, CENP-T, and NDC-80, leading to α-satellite repeat instability [90], mitotic defects, and cell death [80,83,85,91,92,93,94,95].

Interestingly, the activity of the small ubiquitin-like modifier (SUMO)-protease SENP6 was recently found to be required to maintain centromere and kinetochore integrity. SENP6 depletion leads to hyper-SUMOylation of CENP-C and CENP-I, and strong reduction of centromere-bound CENP-C, CENP-T, and CENP-A [96,97]. The KNL-1/Mis12 complex/Ndc80 complex (KMN)-network, NNF1, DSN1, and HEC1 are also reduced after SENP6 depletion [96]. The increase of SUMO chains does not lead to ubiquitin-dependent proteasomal degradation of the CCAN subunits, but to their delocalization [97]. Thus, the entire DNA-associated foundation of the centromere is under the critical control of SENP6 [96].

## 5. Temporal Regulation of CENP-A Deposition: Precision in Action

**Figure 1:**
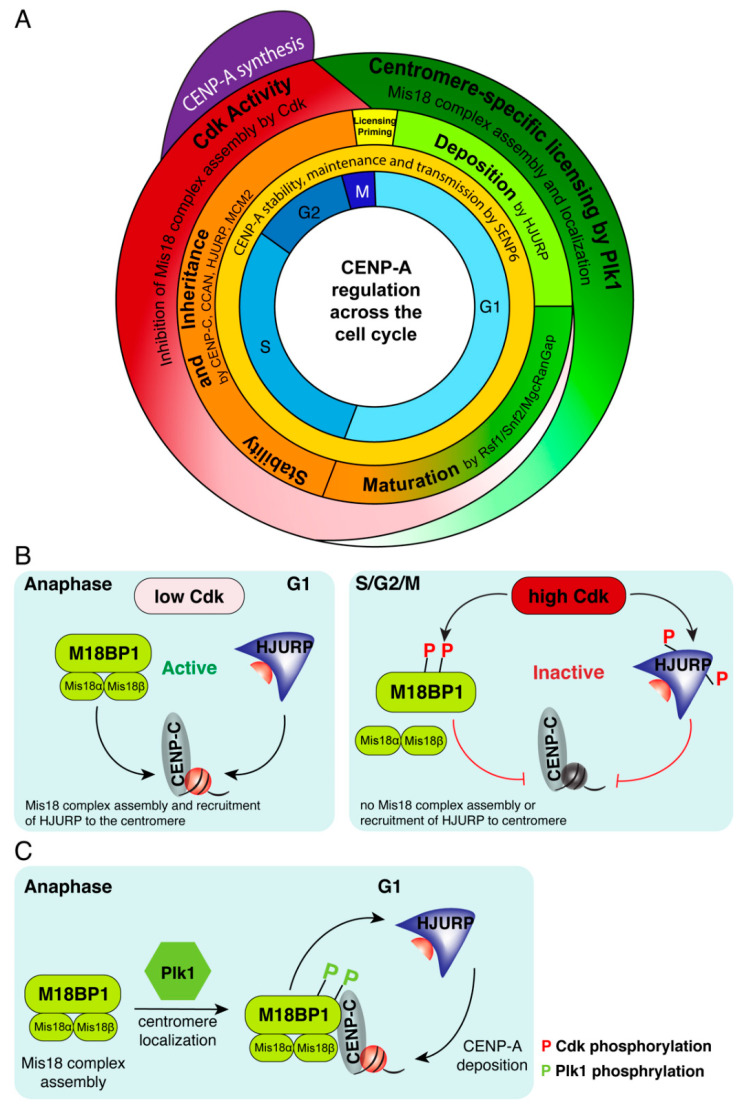
Centromere protein A (CENP-A) deposition and maintenance are cell-cycle dependent. (**A**) CENP-A synthesis, deposition and maturation occur in distinct cell cycle phases and are regulated by coordinated activity of deposition, maturation factors and cell cycle kinases. (**B**) Cyclin-dependent kinase (CDK) regulation of Mis18 complex assembly. Phosphorylation of M18BP1 and Holliday Junction Recognition Protein (HJURP) by CDK1/2 renders them inactive, inhibits assembly of the Mis18 complex, and prevents HJURP recruitment to centromeres, preventing CENP-A deposition. At anaphase, low CDK1/2 levels result in active M18BP1 and assembly of Mis18 complex, recruitment of active HJURP and deposition of new CENP-A at the next G1. (**C**) Phosphorylation of M18BP1 by Plk1 is required for the assembly of the Mis18 complex and its localization to G1 centromeres. Once localized at centromeres by binding to CENP-C, HJURP is recruited to centromeres by binding to the Mis18 complex, leading to the subsequent deposition of new CENP-A.

CENP-A deposition is a tightly regulated process to ensure that CENP-A assembles onto centromeric chromatin only once per cell cycle, although the cell cycle position during which centromeric chromatin is replicated varies in different species [98]. In higher eukaryotes, CENP-A deposition timing is dependent on passage through mitosis [98]. Surprisingly, the replication of centromeric chromatin is uncoupled from centromeric DNA replication. While human centromeric DNA replicates mostly in late S phase [12,99], the deposition and assembly of CENP-A onto the chromatin occurs after exit from mitosis [98,100,101], when its loading chaperone HJURP [102,103] is active [104,105] (Figure 1A). CENP-A assembly is directly linked to cell-cycle progression and is initiated during mitotic exit and restricted to the early G1 phase of the cell cycle. In human cells, HJURP localizes to centromeres at late anaphase/telophase and remains associated with centromeres during G1, the time window during which new CENP-A assembly occurs [102,103].

The targeting of CENP-A and HJURP to centromeres is dependent on the Mis18 complex, comprised of Mis18α, Mis18β and M18BP1. The human Mis18 complex localizes to centromeres at anaphase, just before HJURP, and remains associated until mid G1 [106,107,108,109,110]. Recruitment of Mis18 complex to the centromeres primes them for deposition of CENP-A by HJURP (Figure 1A). Depletion of any subunit of the Mis18 complex, using RNAi, prevents CENP-A deposition, and results in chromosome segregation defects such as misaligned chromosomes and interphase micronuclei [106,111,112]. Mis18α and Mis18β are proposed to form either a heterotetramer [113], or hexamer [114,115] through their C-terminal coiled-coil domains [113]. Mis18BP1 then binds to Mis18α/β and the full complex localizes to centromeres through CENP-C-mediated binding to M18BP1 at anaphase onset [111] (Figure 1C). HJURP is recruited to centromeres by directly interacting with the C-terminal coiled-coil domains of Mis18α/β [113]. Binding of HJURP is proposed to disrupt the Mis18 complex leading to removal of Mis18α [113] and of M18BP1 [105], thereby restricting CENP-A deposition to a single event per cell cycle. Contrarily, recent work proposes that HJURP does not cause dissociation of the Mis18 complex and that HJURP is not dimerized in the process of CENP-A deposition [116], as previously suggested [117]. Deposition of CENP-A:H4 dimers into a homotypic octameric nucleosome is proposed to be achieved through two nearby Mis18:HJURP complexes depositing individual CENP-A:H4 dimers that are tetramerized into the same nucleosome [116].

## 6. Global Regulation Restricts CENP-A Assembly to Early G1

The restriction of CENP-A deposition to early G1 is achieved through global regulation by CDK1/2 activity that occurs during S, G2, and M phase until mitotic exit (Figure 1A,B). CDK1/2 kinases-mediated phosphorylation of two key CENP-A assembly factors, M18BP1 and HJURP, render both inactive, inhibits Mis18 complex assembly and prevents unscheduled centromeric chromatin assembly outside of the G1 phase [104,105] (Figure 1B). At anaphase onset, CDK activity dramatically drops, leading to dephosphorylation of sites within M18BP1 protein [104] (Figure 1A,B). In parallel, polo-like kinase 1 (Plk1)-mediated phosphorylation of Mis18α, Mis18β, and M18BP1 proteins promotes their assembly into the Mis18 complex and its centromeric localization [110,115] (Figure 1C). Thus, Plk1 licenses centromeres for CENP-A deposition by providing a centromere-localization signal for the Mis18 complex. HJURP is subsequently recruited by binding to Mis18α/β [113,118] leading to targeted deposition of new CENP-A at early G1 [100,102,103,110] (Figure 1C).

CENP-A loading at centromeres is mediated by factors that chaperone the loading process. The CENP-A pre-nucleosomal complex was identified in human cells to consist of CENP-A, HJURP, histone H4, NPM1 (nucleophosmin 1) [102,103] and RbAP48 [103]. HJURP, CENP-A’s specific chaperone, binds directly to soluble CENP-A and is required for its chromatin assembly in human [102,103] and xenopus [111]. Depletion of HJURP from human cells [102,103] causes defects in CENP-A assembly [102,103,119]. Furthermore, as mentioned above, tethering HJURP to an ectopic non-centromeric locus is sufficient to induce incorporation of CENP-A into the chromatin at this site and leads to nucleation of a functional *de novo* kinetochore [31,32]. HJURP binds a single CENP-A-histone:H4 heterodimer [120] and is thought to achieve the assembly of homotypic octameric CENP-A nucleosomes via dimerization of HJURP through its C-terminal domain [117] or through two nearby Mis18:HJURP complexes, each depositing individual CENP-A:H4 dimers that are tetramerized into the same nucleosome [116].

## 7. Maturation and Stability of Newly Deposited CENP-A

In the second part of the G1 phase, newly deposited CENP-A goes through incorporation and stabilization processes. This is achieved by the remodeling and spacing factor (RSF) complex [121] and MgcRacGap [122] that interact transiently with centromeres to stabilize newly assembled CENP-A nucleosomes and generate mature centromeric chromatin (Figure 1A). When the cell transitions to S-phase, CDK1/2 activity levels increase, Mis18BP1 is phosphorylated, releases from centromeric chromatin, and CENP-A deposition is inhibited [104,123]. CENP-A synthesis increases in G2 [124,125] (Figure 1A), and newly synthetized CENP-A is bound by the chaperone HJURP in a pre-nucleosomal (non-chromatin associated) complex [102,103]. Increasing CDK1/2 activity inhibits both Mis18BP1 and HJURP (through a cyclin-interacting domain in HJURP) [104,105] and prevents premature CENP-A deposition at G2 phase of the cell cycle (Figure 1B).

The SUMO-protease SENP6, mentioned above, affects strongly not only the entire centromere and kinetochore, but also the assembly and maintenance of CENP-A [96,97]. Loss of SENP6 results in loss of old and newly deposited CENP-A [96], and both Mis18BP1 and Mis18α are regulated by SENP6 [97]. CENP-A is lost from the centromere whether SENP6 is depleted in S, G2, or G1 phase [96]. Thus, SENP6 is continuously required throughout the cell cycle to prevent CENP-A from being removed from the centromere, to stabilize the centromere and kinetochore, and to ensure CENP-A chromatin transmission [96] (Figure 1A).

## 8. CENP-A Inheritance at the Centromeric DNA Replication Fork Crossroad

**Figure 2:**
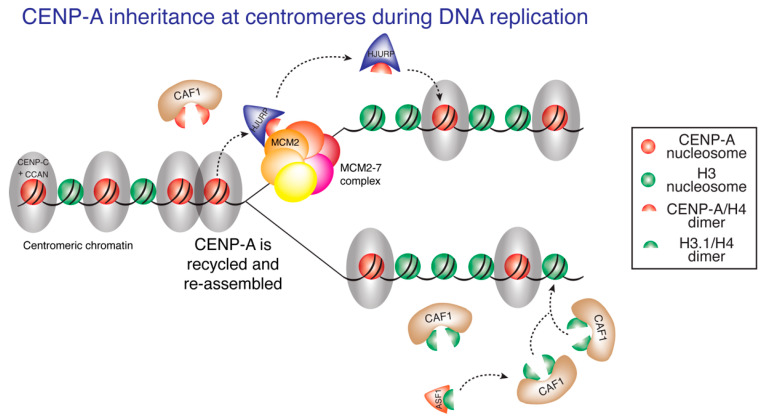
CENP-A inheritance during DNA replication. Presence of the constitutive centromere-associated network (CCAN) complex at centromeric chromatin tethers CENP-A in close proximity to the centromeric DNA replication fork and promotes stable interaction between CENP-A and MCM2. The coordinated action of MCM2, HJURP and CAF1 enables recycling of CENP-A:H4 dimers and their reassembly onto the daughter centromeres, preserving centromere identity.

During centromeric DNA replication, chromatin-bound CENP-A is quantitatively redistributed to each daughter centromere [37,100] and no new CENP-A deposition occurs until early G1. Thus, there is a temporal separation between replication of centromeric DNA and full reconstitution of centromeric chromatin. This raises an important question: how is the centromeric epigenetic mark maintained across the cell cycle, when it would be expected to be displaced by DNA replication and diluted at each centromere, as no new CENP-A is assembled until the next G1 [100]? Using CENP-A ChIP-sequencing and mapping onto centromere reference models for human X chromosome [126] and for each autosome (incorporated into the HuRef genome hg38) [127,128], we recently identified the sequences bound by CENP-A in each of the human centromeres [12]. CENP-A is reproducibly localized to the same centromeric sequences before and after DNA replication. The DNA replication machinery not only replicates DNA but also maintains epigenetically defined centromere identity by mediating precise reassembly of centromere-bound CENP-A chromatin onto the exact same centromeric DNA sequences within the replicating daughter centromere [12] (Figure 2). As mentioned above, CENP-C and the CCAN complex it nucleates are essential for kinetochore assembly prior to mitosis and for ensuring faithful chromosome segregation [80,83,85,91,92,93,94,95]. Surprisingly, CENP-C and the CCAN complex are also essential during DNA replication for the retention of CENP-A at centromeres. Induced rapid degradation of endogenously tagged CENP-C^AID/AID^ at early S-phase, results in a significant loss of CENP-A by G2 [12]. In addition, we find that CENP-A interacts robustly with MCM2 at late S phase, the time of centromeric DNA replication [12]. MCM2 is a core subunit of the DNA replicative helicase MCM2–7 complex that recycles old histones as the replication fork advances [129]. Chromatin assembly factor 1 (CAF1), required for *de novo* chromatin assembly following DNA replication [130], was also copurified with CENP-A in late-S-phase-derived chromatin. These results suggest that CENP-C and the CCAN complex function during centromeric DNA replication to tether CENP-A to the centromeric replication fork, thereby stabilizing CENP-A binding to MCM2 replicative helicase and CAF1 at the time of centromeric DNA replication [12] (Figure 2). At the chromosome arms, MCM2 recycles histone H3/H4 dimers together with the chaperone ASF1 [129,131]. Interestingly, HJURP can interact with MCM2 [129,132] and depletion of HJURP at early S phase results in significant loss of CENP-A from centromeres by G2, demonstrating that recycling of CENP-A:H4 dimers at the centromere by MCM2 requires HJURP as well [132], with HJURP at centromeres perhaps replacing the role of ASF1 at the chromosome arms. Taken together, a model emerges wherein the local CENP-C/CCAN-dependent retention of CENP-A, coupled with the coordinated actions of MCM2, HJURP and CAF1, enables precise reassembly of CENP-A into chromatin within each daughter centromere, thereby maintaining epigenetically defined centromere identity (Figure 2).

Even with retention of CENP-A at the same centromeric sequences, overall quantities of CENP-A are reduced by half, from 200 nucleosomes on average per centromere [37] to 100 nucleosome on average, and human cells undergo mitosis with only half the maximal CENP-A content loaded at centromeres [98,100]. Nucleosomes containing the histone variant H3.3 have been suggested to serve as “placeholders” during S phase in human [133], with subsequent removal in G1, allowing for new CENP-A deposition. A similar placeholder model has been shown to occur in fission yeast with H3 deposition at S phase at centromeres and subsequent removal in G2, when new CENP-A^Cnp1^ is deposited [134].

## 9. DNA Replication Ensures Centromere Specificity

Previous studies have shown that besides assembly at centromeres, a proportion of CENP-A is assembled ectopically onto sites on the chromosome arms [12,14,37,48,135]. Artificially increasing CENP-A expression in human cells increases ectopic deposition at non-centromeric sites, primarily at transcriptionally active sites [12,48], and is accompanied by chromosome segregation aberrations [48,136,137,138]. Therefore, long-term maintenance of centromere identity and function should involve limiting accumulation of non-centromeric CENP-A to ensure the formation of only one centromere per chromosome [12].

**Figure 3:**
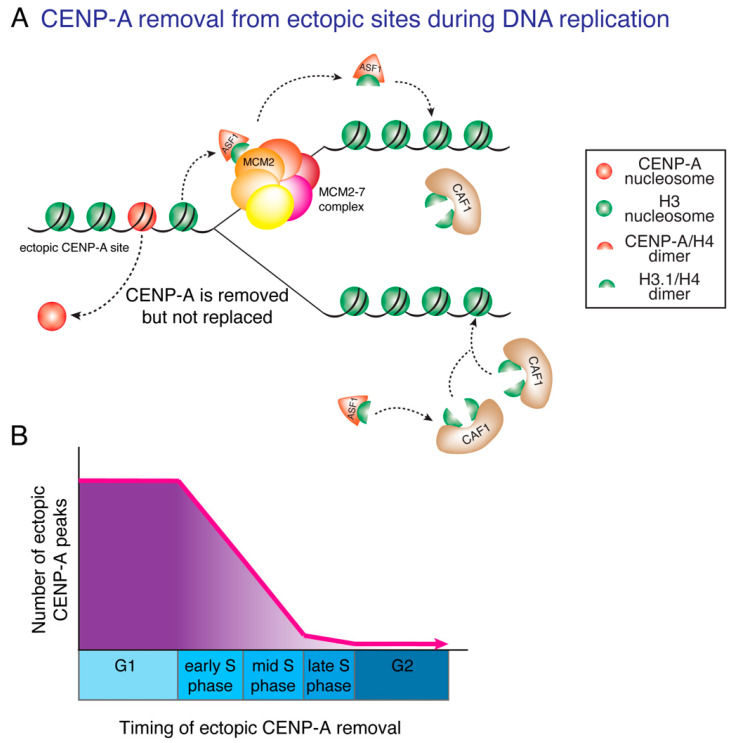
Ectopically loaded CENP-A is removed during DNA replication. (**A**) DNA replication corrects errors in CENP-A deposition by removing ectopic CENP-A from non-centromeric sites. As ectopic CENP-A is not tethered to the replication fork by the CCAN, it is removed from ectopic sites during DNA replication and diluted in the pool of available histones. CENP-A is replaced by histone H3.1:H4 dimers through the combined action of MCM2 and the chaperone ASF1. (**B**) Ectopic CENP-A is primarily found at early- and mid-replicating DNA regions and is removed as replication proceeds during S phase. At the end of S phase, nearly all CENP-A molecules have been removed from ectopic sites.

Although several E3 ligases have been identified that lead to degradation of endogenous CENP-A^Cse4^ [139] and of non-centromeric CENP-A^Cse4^ in budding yeast [140,141,142,143,144], CENP-A^Cnp1^ in fission yeast [145] and CENP-A^CID^ in flies [146,147], almost nothing was known until recently about the pathways that degrade and limit accumulation of non-centromeric CENP-A in humans [123]. Using CENP-A chromatin immunoprecipitation and genome-wide mapping, we recently identified that DNA synthesis not only replicates DNA, but also functions to correct errors in CENP-A deposition by removing ectopic CENP-A from non-centromeric sites [12] (Figure 3A). Most ectopic sites of CENP-A deposition are found in early and mid-replicating regions. As DNA replication progresses, ectopic CENP-A is removed from the replicating chromatin (Figure 3B). In contrast, at the centromere, the same DNA replication machinery mediates the precise reassembly of centromere-bound CENP-A onto the same centromeric DNA sequences, in a coordinated action that requires MCM2 [12,132], HJURP [132], CENP-C and the CCAN complex it nucleates [12] (Figure 2). The concomitant removal of CENP-A from non-centromeric sites and retention of CENP-A at centromeric sites, both achieved during DNA replication, function to maintain epigenetically defined centromere identity, by ensuring that CENP-A is restricted to centromeres only and by preventing accumulation of ectopic CENP-A and acquisition of neocentromeres on the chromosome arms [12].

## 10. Connecting CENP-A, Chromosomal Instability and Cancer

Overexpression of CENP-A has been identified in ~20 different cancer types, including liver, pancreatic, endometrial, breast, ovarian, colorectal, gastric, CNS and lung cancers and is considered a prognostic biomarker of metastatic ability, advanced disease state, poor outcome, and likelihood of relapse ([148,149,150,151,152,153] and Human Protein Atlas). As the defining epigenetic mark of the centromere serving as a keystone for downstream kinetochore assembly, CENP-A is inherently related to maintaining genomic integrity. As such, specific centromere inactivation of the Y chromosome, using CENP-A/histone H3 chimaera that cannot directly recruit CENP-C, leads to high levels of aberrant mitoses followed by similarly high levels of aneuploidy for the Y chromosome [154]. Remarkably, catastrophic numerical and structural chromosomal changes, including chromothripsis (the catastrophic shattering of a single or few chromosomes in a single event), can arise from a single cell division error [154,155,156], that can contribute to cancer development [157]. CENP-A overexpression in cancer may play a role in tumor formation and/or progression through increasing chromosome segregation errors. Indeed, overexpression of CENP-A results in chromosome segregation errors [137], providing a possible mechanism explaining the correlation of CENP-A overexpression with cancer invasiveness and poor prognosis seen in breast adenocarcinoma and lung squamous cell carcinoma [148,158].

Chromosomal instability (CIN), a hallmark of cancer occurring in almost 90% of human tumors [1,159,160], is defined as an increase in the rate of chromosome segregation errors over successive cell divisions, resulting in numerical and structural chromosomal abnormalities [5]. CIN contributes to cancer progression, aggressiveness and ability to evade cancer treatment [5,161]. By causing genomic rearrangements, CIN also contributes to gene expression changes that drive cancer progression [162,163]. CIN can be found early in tumor development and is therefore implicated in cancer initiation and disease progression [2,4,164]. CIN levels correlates positively with tumor stage, likelihood of relapse, prevalence of metastasis and resistance to treatment [5,165].

Interestingly, CIN can originate from mitotic defects caused by lagging chromosomes failing to properly segregate in anaphase, primarily due to incorrect microtubule attachments to the kinetochore [5,159,166]. Such mitotic defects can result in aneuploidy or polyploidy, genomic rearrangements, micronuclei formation, or chromothripsis [3,154,156,167,168]. Levels of CENP-A expression have been shown recently to directly correlate with the number of chromosome segregation defects and incidence of micronuclei formation, linking CENP-A and CIN [137]. However, the complex relationship between CENP-A levels, CIN and gene expression changes in cancer development and/or progression is poorly understood.

## 11. Ectopic Deposition of CENP-A and CIN

**Figure 4:**
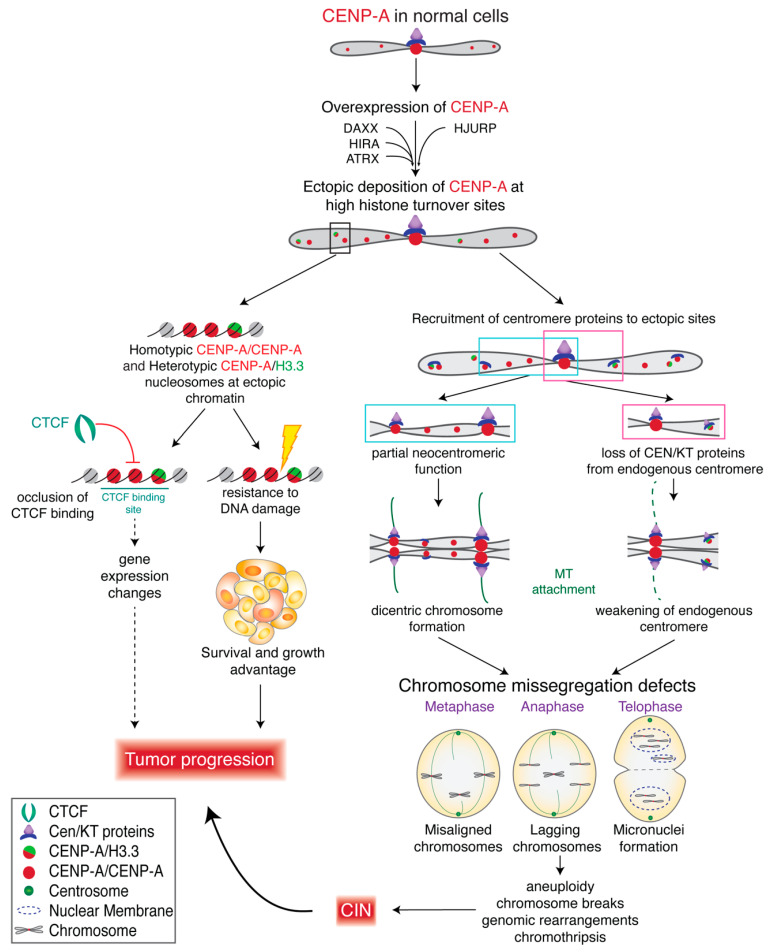
CENP-A overexpression in cancer cells contributes to CIN and tumor progression. Overexpression of CENP-A in cancer cells increases rates of CENP-A ectopic deposition at sites of high histone turnover. Ectopic CENP-A, consisting of homotypic or heterotypic particles, can occlude CCCTC-binding factor (CTCF) binding and regulatory elements which may contribute to gene expression changes that further affect tumor progression. CENP-A overexpression can also confer survival advantage to tumors by increasing resistance to DNA damage. CENP-A overexpression may recruit centromere and kinetochore (CEN/KT) proteins to ectopic CENP-A sites and can trigger formation of neocentromeres, while weakening endogenous centromeres and leading to chromosome segregation defects.

The primary mechanism connecting CENP-A and CIN is the mislocalization of CENP-A-containing nucleosomes to ectopic positions on chromosome arms, away from the centromere [12,14,48,135,137]. Ectopic deposition of CENP-A has been shown to occur naturally in RPE-1 (chromosomally stable diploid retinal pigmented epithelial) cells [37] and accounts for ~73% of total chromatin-bound CENP-A, measured using several quantitative fluorescence-based methods [37]. Using CENP-A ChIP-sequencing, 17% [48] to 25% [12] of CENP-A-bound DNAs are reported to be non-centromeric in HeLa (immortalized cervical cancer) cells expressing endogenous levels of CENP-A. Upon overexpression of CENP-A in HeLa cells (4–5× fold), non-centromeric CENP-A-bound DNAs increase to ~50% of total CENP-A-bound DNAs [12,14,48]. An increase in ectopic deposition upon CENP-A overexpression has been demonstrated in HeLa cells [12,48,137], DLD1 (colorectal adenocarcinoma) cells [12], RPE-1 cells [137] and SW480 (colorectal cancer) cells [135], using immunofluorescence, CENP-A ChIP-sequencing and CENP-A ChIP-qPCR. Most importantly, mislocalization of overexpressed CENP-A to the chromosome arms in HeLa and RPE-1 cells resulted in chromosome congression defects, lagging chromosomes, micronuclei formation and a delay in mitotic exit, directly linking CENP-A overexpression and ectopic CENP-A deposition with CIN [137]. This was associated with altered localization of CENP-C, CENP-T and Nuf2 and weakened native kinetochores [138] (Figure 4).

HJURP, CENP-A’s chaperone, deposits CENP-A at centromeres [102,103] at exit of mitosis [100,101], resulting in assembly of CENP-A into a homotypic octameric nucleosome with two molecules of CENP-A [14,48,51,52,57]. When overexpressed, CENP-A has been suggested to form, in addition to the homotypic nucleosome, a heterotypic octameric particle containing one molecule of CENP-A and one molecule of histone H3.3, that is deposited at ectopic sites by the histone H3.3 chaperone death domain-associated protein 6 (DAXX) [48,137]. While centromeric CENP-A particles were largely homotypic, those in chromosome arms were reported to be made up of both types of particles [48] (Figure 4). Importantly, DAXX is also upregulated in some cancer cell lines where CENP-A is upregulated [135], and both DAXX and HJURP have structurally similar histone binding domains [169]. Notably, siRNA-mediated depletion of DAXX in HeLa cells overexpressing CENP-A prevents ectopic deposition of CENP-A [48] and suppresses chromosome segregation defects [137], suggesting a role for DAXX in ectopic CENP-A deposition and linking CENP-A expression levels to CIN. Interestingly, fly CENP-A^CID^ has been shown to use a separate loading mechanisms for its incorporation into centromeric and ectopic sites; CAL1 is required for loading at the centromere, while the NuRD chromatin remodeling complex is required for ectopic CENP-A^CID^ incorporation [170]. A possible role in depositing CENP-A at non-centromeric sites was also suggested for ATRX [135] and HIRA [171].

**Figure 5:**
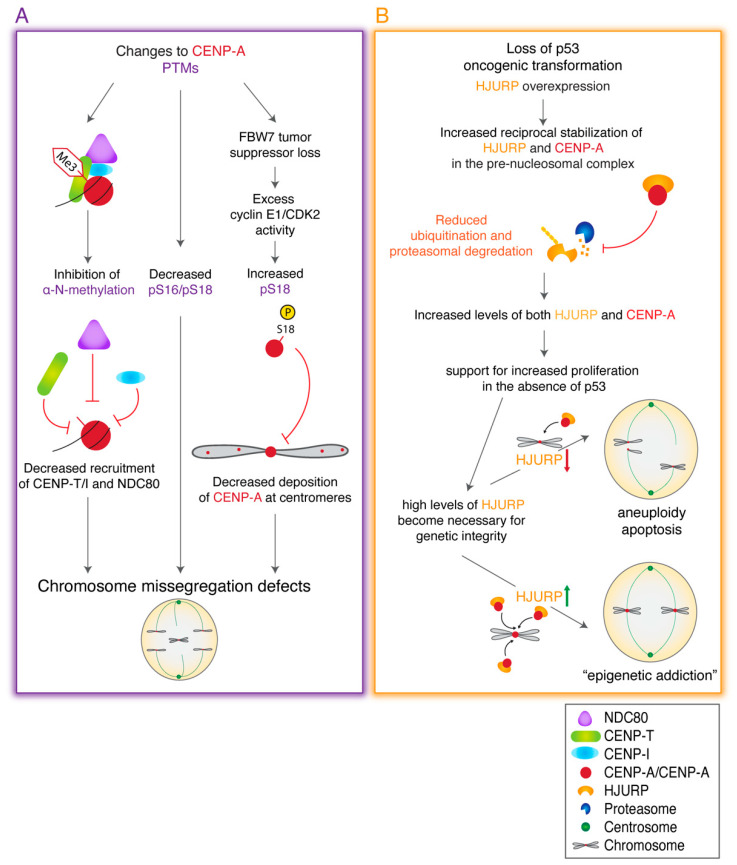
Alterations to CENP-A PTMs and/or to HJURP expression levels contribute to CIN and tumor progression. (**A**) Alterations to CENP-A posttranslational modifications (PTMs) can reduce recruitment of CCAN and kinetochore components, reduce CENP-A deposition at centromeres, and lead to chromosome segregation defects. (**B**) HJURP overexpression stabilizes CENP-A in the pre-nucleosomal complex and CENP-A protects HJURP from degradation. In p53 null cancers, upregulated HJURP levels become critical for maintaining centromere integrity and preventing apoptosis as p53-mediated DNA checkpoints are lost, leading to HJURP epigenetic addiction that contributes to tumor survival and progression.

Studies from Lacoste and colleagues [48] and from us [12,14] have used HeLa cells exhibiting similar levels of CENP-A overexpression (4.5× fold) and found similar levels (~50%) of CENP-A mislocalization. Nevertheless, our data shows that the overwhelming majority of human CENP-A chromatin particles, in these overexpression conditions, are octameric nucleosomes containing two molecules of CENP-A at all cell cycle points, with heterotypic CENP-A/histone H3-containing nucleosomes comprising at most 2% of total CENP-A-containing chromatin [14]. Thus, the overwhelming majority of ectopic CENP-A-containing chromatin is assembled with homotypic CENP-A nucleosomes in our CENP-A overexpressing HeLa cell line (Figure 4), suggesting that HJURP might still play a significant role in ectopic deposition of CENP-A. Importantly, HJURP is also elevated in certain cancers (Figure 5B), including breast and pancreatic cancers, where upregulation of HJURP was found to correlate with decreased survival [172], and HJURP expression level serves as an unfavorable prognostic marker in melanoma, as well as pancreatic, liver, and lung cancers ([173,174], and The Cancer Genome Atlas (TCGA) data).

## 12. CENP-A Misregulation and Gene Expression

Ectopic deposition of CENP-A may have consequences beyond competing with and weakening native centromeres. CENP-A nucleosomes confer unique properties to chromatin where they are deposited, resulting in DNA unwinding of the entry/exit sites of the nucleosome [14,48,57] and producing a more globally condensed chromatin state [58]. Deposition of CENP-A into ectopic sites might therefore affect DNA accessibility, chromatin secondary structure, and gene expression (Figure 4). Consistent with this idea, CENP-A molecules deposited on chromosome arms are found primarily in sites of high histone turnover, such as active transcription sites, regulatory regions, and sites of CTCF binding [12,48,135], leading to occlusion of CTCF binding [48]. Occlusion of CTCF binding has been shown in many cancer types, is associated with aberrant gene expression and is thought to contribute to tumorigenicity [175,176,177,178]. Surprisingly, no significant gene expression changes were identified in HeLa cells showing ectopic CENP-A mislocalization and occlusion of CTCF binding sites [48]. It remains to be established whether gene expression is affected at higher levels of CENP-A overexpression and/or in different types of cancer.

In SW480 colorectal cancer cells CENP-A hotspots accumulate at subtelomeric chromosomal locations, including at the 8q24/Myc region long-associated with genomic instability, and with CENP-C recruitment to this location. CENP-A deposition at this locus occurs in early stage colorectal tumors [135,171] and is correlated with amplification and overexpression of the MYC gene within that locus [171].

Lastly, overexpression of CENP-A alone, regardless of its deposition mechanics, appears to have some effect on gene expression dynamics. Hepatocellular carcinoma cells overexpressing CENP-A exhibit elevated expression of p53 inhibitor MDM2 and anti-apoptotic Bcl-2, decreased expression of pro-apoptotic Bax and modulate numerous cell cycle regulators [179]. Moreover, analyses of gene expression data from numerous primary cancer cell lines and patient clinical samples have identified 14 centromere and kinetochore assembly genes that are consistently overexpressed in human cancers [158]. These 14 genes include regulators of CENP-A nucleosome assembly (HJURP, Mis18β), centromere proteins (CENP-A, -K, -L, -M, -N, -U, -W), and kinetochore proteins (NDC80, SPC24, SPC25, NUF2, ZWINT), highlighting the role of centromere and kinetochore gene misexpression in cancer progression [158]. Thirty-nine percent of the 18 cancer types analyzed showed significant positive correlation between the 14 CEN/KT elevated genes signature and genome instability (measured by copy-number alterations (CNA) and mutation frequency), including breast, lung and stomach adenocarcinomas (ADC) and low-grade brain gliomas. Moreover, this 14 CEN/KT elevated genes signature also correlated with poor patient survival for breast and lung cancers [158].

## 13. Perturbations in CENP-A Posttranslational Modifications are Linked to Cancer

CENP-A function is also regulated by posttranslational modifications to the CENP-A histone itself. Several sites of posttranslational modification have been identified for CENP-A, including α-amino trimethylation [180], Ser7 phosphorylation [181,182], Ser16/18 phosphorylation [161,183], Ser68 phosphorylation [184,185], Lys124 ubiquitination [185,186,187,188], Lys124 acetylation [50,189], and Lys124 monomethylation [189], some of which are emerging as important regulators of CENP-A deposition and function within the centromere [reviewed at Srivastava and Foltz [190]].

Several perturbations in CENP-A posttranslational modifications have been linked to chromosome segregation defects and cancer (Figure 5A). Inhibition of CENP-A α-amino trimethylation causes a reduction in CENP-T and CENP-I at the centromere, leading to lagging chromosomes [180]. Furthermore, CENP-A is known to be phosphorylated at two highly conserved residues, Ser16 and Ser18 (located within the amino terminal tail of CENP-A), prior to its deposition and in the CENP-A nucleosome [183]. Loss of phosphorylation at Ser16/18 has been shown to lead to chromosome missegregation [183]. In contrast, hyper-phosphorylation at Ser18 also lead to chromosome missegregation and severe CIN phenotype and is linked to the loss of FBW7 [161], an E3 ubiquitin ligase and one of the most frequently lost tumor suppressors in human cancers [191]. CENP-A Ser18 is a substrate for cyclinE1/CDK2 phosphorylation and is cell cycle regulated [161]. Hyperphosphorylation of CENP-A Ser18 by cyclinE1/CDK2 reduced its centromeric localization, increased levels of lagging chromosomes, chromosomal bridges, and micronuclei formation, and even promoted anchorage-independent growth and xenograft tumor formation [161]. Thus, cyclin E1/CDK2 activation coupled with tumor suppressor FBW7 loss can promote CIN and tumor progression through reducing CENP-A’s centromeric localization, leading to centromere dysfunction (Figure 5A).

## 14. CENP-A Overexpression May Be Indispensable for Cancer Progression

Typically, CENP-A and HJURP protein levels are tightly co-regulated in accordance with cell cycle progression [103,123] and *CENPA* and *HJURP* genes have similar CDE/CHR binding motifs in their promoters, suggesting that they are both negatively regulated indirectly by p53 through the DREAM complex [138] and/or through FOXM1 [192,193]. Consistent with that, both CENP-A and HJURP are transcriptionally up-regulated in p53-null human tumors [138]. In addition, CENP-A and HJURP have reciprocal stabilizing effects on each other. Specifically, siRNA-mediated depletion of CENP-A promote proteasome-dependent degradation of HJURP [138] and HJURP stabilizes CENP-A [102,103]. In flies, CAL1 mediates CENP-A^CID^ ubiquitylation by CUL3/RDX that stabilizes CENP-A^CID^ and CAL1 [194]. When expression of either CENP-A or HJURP is reduced in non-cancerous cells, deposition of new CENP-A at exit of mitosis is diminished, causing loss of centromere identity and increased segregation errors and micronuclei [26,41,102,103]. Additionally, degradation of HJURP in early S phase impairs retention of CENP-A at centromeres throughout S phase [132]. All of this demonstrates that co-regulation of CENP-A and HJURP is important for maintaining a fine balance that preserves centromere identity.

As discussed previously, CENP-A is upregulated in many cancers and correlates with multiple markers of poor prognosis [148,158]. Like CENP-A, HJURP is overexpressed in certain cancers, including breast, liver, pancreatic, lung and melanoma cancers (The Human Protein Atlas), where upregulation of HJURP correlates with decreased survival [172]. Furthermore, partial depletion of CENP-A by about 50–70% in cancer cells overexpressing CENP-A reduces proliferation and cell cycle progression, colony formation, migration and invasiveness, and increases rates of apoptosis [179,195]. Similarly, in cancer cells overexpressing HJURP, 80% depletion of HJURP using siRNA [196], or complete knockout of HJURP using CRISPR/Cas9 [138], induces cell cycle arrest and apoptosis, recapitulating the phenotype of CENP-A depletion in cancer cells. Though HJURP loss in wild-type cells causes cell cycle arrest, in p53-null transformed or cancer cells, HJURP loss leads to aneuploidy and elevated rates of apoptosis [138].

Interestingly, CENP-A and HJURP are both elevated following p53 loss and oncogenic transformation in what is proposed to be an “epigenetic addiction”, where the transformed cells require elevated HJURP for growth and survival [138] (Figure 5B). In this model, HJURP and CENP-A levels are increased upon p53 loss and increase further following oncogenic transformation. In cells with p53, depletion of HJURP causes slow depletion of CENP-A at centromeres that can be sensed by p53 resulting in cell cycle arrest and maintenance of genome integrity. In cells lacking p53, HJURP depletion results in rapid CENP-A loss at centromeres, as the cells divide quickly. However, the loss of p53 prevents sensing of CENP-A loss, leading to severe centromere dysfunction, aneuploidy and apoptosis [138]. Together, these data highlight that overexpression of CENP-A and HJURP is essential for maintaining centromere identity and promoting survival in cancer cells, particularly as loss of p53 and cell cycle checkpoints allow rapid cell cycle progression without sensing of genome integrity. Cancer cells become “addicted” and require the sufficient expression of CENP-A and HJURP to ensure centromere identity is maintained and chromosome segregation is achieved/protected in the face of widespread genomic instability (Figure 5B).

In summary, the roles of CENP-A in cancer progression are multifaceted and complex. CENP-A overexpression, which is commonly observed in various cancers, can increase the rates of ectopic deposition of CENP-A through the collaborative efforts of multiple histone chaperones. Subsequently, sites of ectopic CENP-A may acquire partial neocentromeric function, weakening endogenous centromeres and contributing to increased occurrence of CIN. Alteration to posttranslational modifications (PTMs) of CENP-A in cancer reduce centromeric CENP-A deposition and recruitment of CCAN and kinetochore proteins, further destabilizing the centromere and favoring CIN. Ectopic CENP-A can also occlude binding of CTCF, a regulator of gene expression. Additionally, overexpression of CENP-A confers resistance to DNA damage across the genome [48], which could provide a survival advantage to tumors. CENP-A overexpression can also be accompanied by overexpression of its primary chaperone, HJURP, which increases the available pool of CENP-A for deposition at centromeres and possibly at ectopic sites. p53 loss, a key step in oncogenic transition, leads to overexpression of both CENP-A and HJURP and they become indispensable for maintaining centromere identity. Although currently unexplored, CENP-A is a potential target for modulating CIN as a therapeutic strategy in cancers. Targeting overall levels of CENP-A or perhaps ectopically loaded CENP-A specifically may provide a therapeutic benefit for cancers overexpressing CENP-A.
